# The role of discoidin domain receptor 2 in the renal dysfunction of alport syndrome mouse model

**DOI:** 10.1080/0886022X.2021.1896548

**Published:** 2021-03-11

**Authors:** Yuya Sannomiya, Shota Kaseda, Misato Kamura, Hiroshi Yamamoto, Hiroyuki Yamada, Masataka Inamoto, Jun Kuwazuru, Saki Niino, Tsuyoshi Shuto, Mary Ann Suico, Hirofumi Kai

**Affiliations:** a Department of Molecular Medicine Graduate School of Pharmaceutical Sciences, Kumamoto, Japan; b Program for Leading Graduate Schools “HIGO (Health life science: Interdisciplinary and Glocal Oriented) Program”, Kumamoto University, Kumamoto, Japan; c Ono Pharmaceutical Co. Ltd, Osaka, Japan; d Global Center for Natural Resources Sciences, Faculty of Life Sciences, Kumamoto University, Kumamoto, Japan

**Keywords:** Alport syndrome, discoidin domain receptor 2 (DDR2), Type IV collagen, proteinuria, inflammatory cytokines, fibrosis

## Abstract

Alport syndrome (AS) is a hereditary glomerular nephritis caused by mutation in one of the type IV collagen genes α3/α4/α5 that encode the heterotrimer COL4A3/4/5. Failure to form a heterotrimer due to mutation leads to the dysfunction of the glomerular basement membrane, and end-stage renal disease. Previous reports have suggested the involvement of the receptor tyrosine kinase discoidin domain receptor (DDR) 1 in the progression of AS pathology. However, due to the similarity between DDR1 and DDR2, the role of DDR2 in AS pathology is unclear. Here, we investigated the involvement of DDR2 in AS using the X-linked AS mouse model. Mice were treated subcutaneously with saline or antisense oligonucleotide (ASO; 5 mg/kg or 15 mg/kg per week) for 8 weeks. Renal function parameters and renal histology were analyzed, and the gene expressions of inflammatory cytokines were determined in renal tissues. The expression level of DDR2 was highly elevated in kidney tissues of AS mice. Knockdown of *Ddr2* using *Ddr2*-specific ASO decreased the *Ddr2* expression. However, the DDR2 ASO treatment did not improve the proteinuria or decrease the BUN level. DDR2 ASO also did not significantly ameliorate the renal injury, inflammation and fibrosis in AS mice. These results showed that *Ddr2* knockdown by ASO had no notable effect on the progression of AS indicating that DDR2 may not be critically involved in AS pathology. This finding may provide useful information and further understanding of the role of DDRs in AS.

## Introduction

Alport syndrome (AS) is a hereditary disease that causes progressive loss of kidney function. It is caused by a mutation in one of the type IV collagen genes that code for COL4A3, COL4A4, and COL4A5 proteins. These type IV collagens form protomer network that are important components of the glomerular basement membrane (GBM). Mutations in type IV collagen genes disrupt the structure and function of GBM [1,2]. Abnormal GBM structure interferes with the glomerular filtration system leading to proteinuria, inflammation, renal fibrosis and finally to end-stage renal disease (ESRD). Currently, renin angiotensin aldosterone system (RAAS) inhibitors are used for AS therapy. Although early treatment with RAAS inhibitor delays renal failure in AS patients, the disease eventually progresses into ESRD [3,4]. It is now generally believed that RAAS inhibitor is not sufficient for AS therapy. Therefore, it is important to continue the search for novel therapeutic targets that have different mechanism from RAAS inhibitors.

Several tyrosine kinase receptors signaling such as epidermal growth factor receptor (EGFR) and integrin are activated in AS mouse model. Treatment with erlotinib, an EGFR inhibitor, suppressed the expression of renal inflammatory cytokines in *Col4a5* mutant AS mice [5]. Furthermore, lack of *Itga2* gene, the α2 subunit of integrin receptor, ameliorated the disease progression in *Col4a3*^-/-^ AS mouse model [6]. These reports indicated that tyrosine kinase and collagen receptors could be new therapeutic targets of AS. Discoidin domain receptors (DDRs) are tyrosine kinase receptors that have characteristic extracellular discoidin element and bind various types of collagen. DDRs are activated by collagens independently from integrins [7]. DDRs have two isoforms, DDR1 and DDR2. DDR1 is mainly expressed in epithelial cells and activated by types I, II, III, IV, and XI collagen, while DDR2 is expressed in mesenchymal cells and activated mainly by types I-III and X collagen [8,9]. Previous report indicated that the expression of *Ddr1* gene is upregulated in glomerulonephritis, and the knockout of *Ddr1* gene ameliorated renal pathology in a mouse model of glomerulonephritis [10]. Moreover, lack of *Ddr1* gene delayed the disease progression in *Col4a3*^-/-^ AS mouse model [11], and pharmacological DDR1 inhibition suppressed albuminuria and renal fibrosis in *Col4a3*^-/-^ mice [12]. These reports suggested that DDR1 is a potential gene target for AS therapy. But because DDR1 and DDR2 have high homology [9], whether DDR2 is associated with AS progression needs to be investigated. Presently, it is unclear what the role of DDR2 is in the pathology of AS.

In this study, we found that *Ddr2* mRNA expression was elevated in the kidney of the AS mouse model *Col4a5* G5X in which glycine at codon 5 is mutated to a stop codon. We then examined the effect of *Ddr2* knockdown using antisense oligonucleotide (ASO). We found that treatment with DDR2 ASO only tended to slightly decrease a few inflammatory cytokines such as *Il-1b*, *Il-8* and *Mcp1*, and a pro-fibrotic gene *Col1a1*; but it did not ameliorate renal dysfunction in AS mice. These results indicate that DDR2 is not associated with disease progression of AS. This finding may provide useful information and further understanding of the role of DDRs in AS.

## Materials and methods

### Animals and *in vivo* treatment

An X-linked AS mouse model (*Col4a5^tm1Yseg^* G5X mutant; a missense mutation that converted Gly codon 5 to a Stop codon) was described previously [13]. These mice were obtained from the Jackson Laboratory (Bar Harbor, USA). Age-matched wild-type (WT) C57BL/6 mice (Charles River Laboratories) were used in experiments as control to compare with AS mice. Mice were housed in clean vivarium and fed with food and water *ad libitum*. In all experiments, male mice were used to eliminate sex difference due to sex-linked inheritance of *Col4a5tm1Yseg* G5X mutation. Sixteen-week-old WT and AS mice were treated with saline or antisense oligonucleotide (ASO, 5 mg/kg or 15 mg/kg, subcutaneously, per week) for 8 weeks. ASO was gifted by Ono Pharmaceutical Corporation (Osaka, Japan). All animal experiments were approved by the Animal Care and Use committee of Kumamoto University, Kumamoto, Japan (Permit number: I29-199).

### Antisense oligonucleotide

Antisense oligonucleotides in this study were produced according to the custom design generated by Exiqons proprietary design software for optimal performance. Control antisense oligonucleotide (CON ASO; Antisense LNA GapmeR *in vivo* large scale, ID: 230748709) is a 15-nucleotide-long gapmer with the following sequence: AACACGTCTATACGC, and DDR2 antisense oligo (DDR2 ASO; Antisense LNA GapmeR *in vitro* Premium, ID: 588952-2) is a 16-nucleotide-long gapmer with the following sequence: GACATCTAGTGCAAAA. Both oligonucleotides have phosphorothioate backbone. These oligonucleotides were analyzed by anion-exchange high performance chromatography, desalted and lyophilized as a sodium salt for CON ASO, or by high-performance liquid chromatography (HPLC) for DDR2 ASO. The identity of these compounds was confirmed by electrospray ionization (ESI)-mass spectrometry (MS) for CON ASO and MS for DDR2 ASO.

### Proteinuria score

Mouse urine samples were collected for 24 h once every two weeks using metabolic cage (AS ONE Corporation, Osaka, Japan). Urinary protein and creatinine were measured by Bradford method (Bio-Rad, CA, USA) and Jaffe’s method (Wako Pure Chemicals, Osaka, Japan), respectively, as described previously [14]. Urinary protein concentration was normalized with urinary creatinine concentration, and presented as proteinuria score.

### Plasma creatinine and blood urea nitrogen

Mouse blood samples were obtained from abdominal aorta. Fresh blood samples were centrifuged at 3000 rpm, 4 °C, for 15 min, and blood plasma was collected. Serum creatinine was measured by Jaffe’s method (Wako Pure Chemicals). Blood urea nitrogen (BUN) of plasma was measured using Urea Nitrogen B Test (Wako Pure Chemicals). The analyses of samples were carried out according to the manufacturer’s recommended protocol.

### Renal histology and evaluation of glomerular injury score

For histological analysis, mouse renal tissues were fixed in 10% formalin, followed by 70% ethanol dehydration, embedded in paraffin and sectioned at 4-µm or 2-µm thickness for periodic acid-Schiff (PAS), periodic acid-methenamine silver (PAM) and Masson-trichrome staining. Glomerular injury score was assessed as described previously [15]. More than 100 PAS-stained random glomeruli per mouse (*n* = 5–6 mice) were examined, and scored from 0 to 4 (0, no lesion; 1, expansion of mesangial area; 2, expansion of Bowman’s epithelial cells and adhesion of glomeruli and Bowman’s capsule; 3, sclerotic area in 50–75% of glomerulus; 4, sclerotic area in 75–100% of glomerulus). Scoring was performed using Bio-Revo imaging and analysis software (Keyence, Japan). Values were computed and presented in a graph as percentage.

### Real-time RT-PCR analysis

Total RNA was isolated from kidneys using RNAiso Plus (Takara Bio Inc, Japan) with homogenization according to the manufacturer’s recommendation. Quantitative RT-PCR (qRT-PCR) was carried out on the purified RNA using SYBR Green Master Mix (Applied Biosystems). Reverse transcription and PCR amplifications were performed as described previously [16]. The sequences of primers used for qRT-PCR are shown in Table 1.

**Table 1.** Primer sequences for real-time PCR.

Gene	Sense	Antisense
*Ddr1*	5′-CTCCACCCCATTCTGCAC-3′	5′-CAGAAGGAGGCGGTAGGC-3′
*Ddr2*	5′-ATCACAGCCTCAAGTCAGTGG-3′	5′-TTCAGGTCATCGGGTTGCAC-3′
*Lysozyme*	5′-CCAGTGTCACGAGGCATTCA-3′	5′-TGATAACAGGCTCATCTGTCTCA-3′
*Lcn2*	5′-CAGAAGGCAGCTTTACGATG-3′	5′-CCTGGAGCTTGGAACAAATG-3′
*Il-1β*	5′-GCTGAAAGCTCTCCACCTCAATG-3′	5′-TGTCGTTGCTTGGTTCTCCTTG-3′
*Il-6*	5′-GAGGATACCACTCCCAACAGACC-3′	5′-AAGTGCATCATCGTTGTTCATACA-3′
*KC (Il-8)*	5′-TGTCAGTGCCTGCAGACCAT-3′	5′-CCTCGCGACCATTCTTGAGT-3′
*Mcp1*	5′-GAAGCTGTAGTTTTTGTCACCAAG-3′	5′-AGGTAGTGGATGCATTAGCTTCA-3′
*α-Sma*	5′-CCCAGACATCGAGGAGTAATGG-3′	5′-TCTATCGGATACTTCAGCGTCA-3′
*Tgfβ*	5′-CACCTGCAAGACCATCGACAT-3′	5′-GAGCCTTAGTTTGGACAGGATCTG-3′
*Col1a1*	5′-CTGGCGGTTCAGGTCCAAT-3′	5′-TTCCAGGCAATCCACGAGC-3′
*Gapdh*	5′-CCTGGAGAAACCTGCCAAGTATG-3′	5′-GGTCCTCAGTGTAGCCCAAGATG-3′

### Western blotting

Mouse kidneys were isolated and lysed in radio immunoprecipitation (RIPA) buffer [50 mM Tris-HCl, 150 mM NaCl, 1 mg/ml sodium deoxycholate, and 1% NP-40, containing 1% protease inhibitor cocktail (Sigma-Aldrich, MO, USA), 2 mM sodium vanadate and 100 mM sodium fluoride] [17]. Protein lysates were subjected to SDS-PAGE analysis. Immunoblots were probed with DDR1 (D1G6), DDR2 (clone 2B12.1), phosphorylated ERK (p-ERK; #4370), ERK (#4695) and Vinculin (ab129002) antibodies. DDR1, p-ERK and ERK antibodies were from Cell Signaling Technology (MA, USA). DDR2 was from Merck, (NJ, USA). Vinculin was from Abcam (Cambridge, UK). ECL-Prime Western Blotting Detection Reagents and ECL Western Blotting Analysis system (Amersham, UK) were used for visualizing the blots.

### Statistical analysis

All the data are presented as mean ± SE The statistical significance of the difference between two groups was assessed using Student’s *t* test. For more than two-group comparison, statistical difference among the groups was analyzed using one-way analysis of variance (ANOVA) with Dunnett’s test as indicated in the figure legends. Statistical analysis was performed using JMP13 Statistical Discovery^TM^ (SAS Institute Inc., Cary, NC, USA). A *p* value <.05 is considered statistically significant.

## Results

### *Ddr2* mRNA expression level increases with progression of as in mice

**Figure 1.**
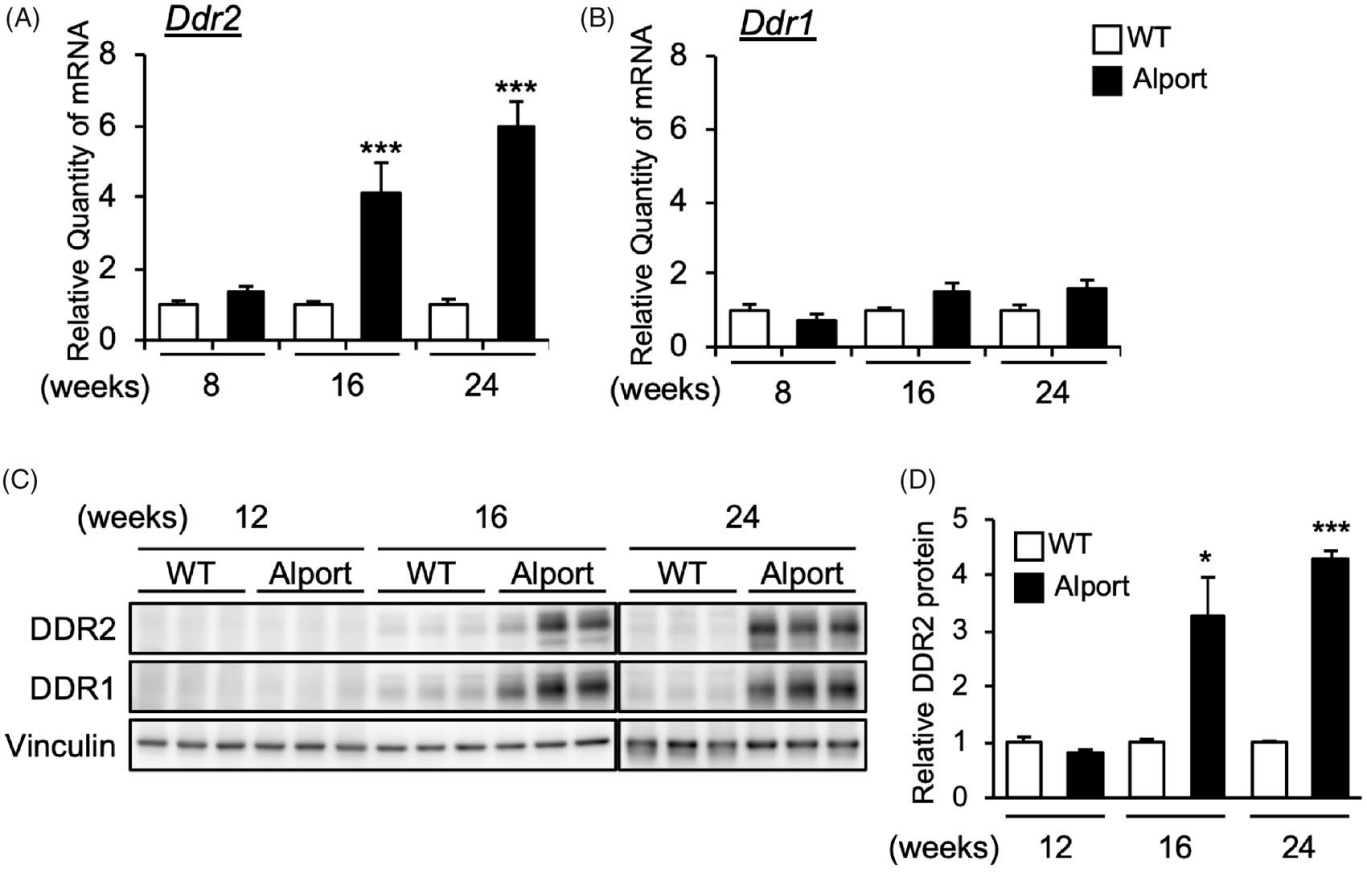
Expression of DDR2 in kidneys of Alport mice. (A,B) Total RNA was isolated from kidney tissues of 8-, 16-, and 24-week-old wild-type (WT) or Alport syndrome mice. Quantitative RT-PCR was performed to evaluate the expression of *Ddr2* and *Ddr1*. The data were normalized to *Gapdh*. Bars indicate the mean ± S.E. (*n* = 4–6). ***p<.001 vs WT, assessed by Student’s *t* test. (C) Immunoblots of protein lysates from whole kidneys of WT and Alport mice were probed with the indicated antibodies. (D) The DDR2 expression was quantified by multi gauge software and normalized with Vinculin (loading control). Bars indicate the mean ± S.E. (*n* = 3). *p<.05, ***p<.001 vs WT, assessed by Student’s *t* test.

We examined the expression of *Ddr2* and *Ddr1* genes in AS mice kidneys, and found that *Ddr2* (Figure 1(A)) but not *Ddr1* (Figure 1(B)) mRNA level was significantly elevated in AS mice in a time-dependent manner. Specifically, *Ddr2* mRNA was significantly increased in the kidneys of 16- and 24-week-old AS mice (Figure 1(A)). Consistently, DDR2 protein level was also increased in AS mice kidneys at 16 and 24 weeks old (Figure 1(C,D)). These results showed that DDR2 expression was increased with the progression of Alport syndrome. Interestingly, DDR1 protein level was also highly increased in 16- and 24-week-old AS mice (Figure 1(C)) although *Ddr1* mRNA level was only slightly elevated (Figure 1(B)).

### DDR2 ASO specifically inhibits DDR2 expression in as mice

**Figure 2.**
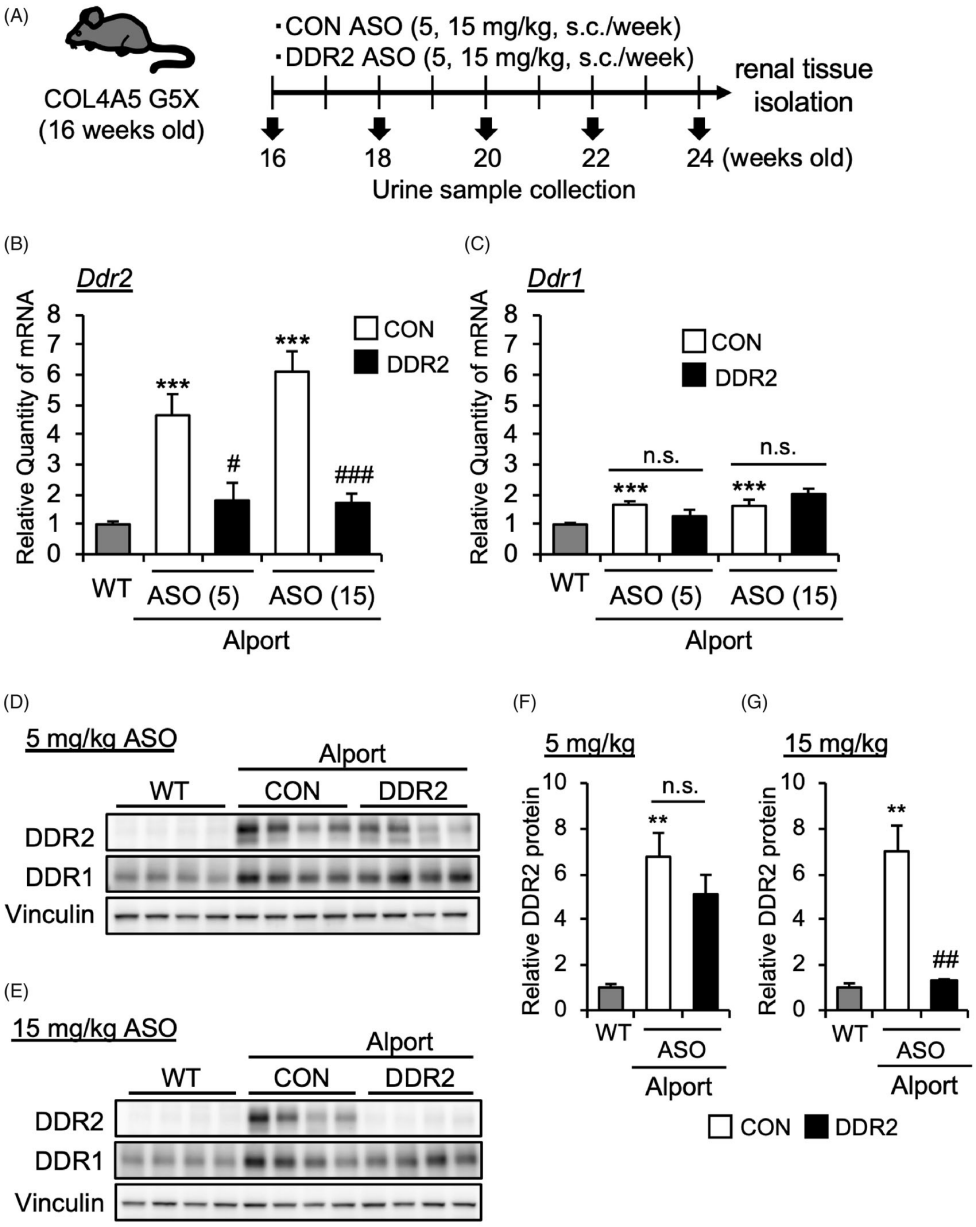
DDR2 ASO inhibits DDR2 expression specifically in Alport mice. (A) Scheme of experimental plan for CON ASO and DDR2 ASO injection. (B,C) Total RNA was isolated from kidney tissues of 24-week-old mice. Quantitative RT-PCR was performed to evaluate the expression of *Ddr2* and *Ddr1*. The data were normalized to *Gapdh*. Bars indicate the mean ± S.E. (*n* = 5–6). ***p<.001 vs WT; ^#^*p*<.05, ^###^*p*<.001 vs CON ASO, assessed by Dunnett’s test. n.s., not significant. (D,E) Immunoblotting of protein lysates from whole kidney of WT, CON ASO- and DDR2 ASO-injected mice. (F,G) Blots of DDR2 were quantified by multi gauge software and normalized with Vinculin (loading control). Bars indicate the mean ± S.E. (*n* = 4). **p<.01 vs WT; ^##^*p*<.01 vs CON ASO, assessed by Dunnett’s test.

Our data above suggested that DDR2 was activated in AS mice especially starting from 16 weeks of age. To determine the contribution of DDR2 in Alport syndrome pathology, we treated 16-week-old AS mice with DDR2 antisense oligonucleotide to inhibit DDR2 expression. DDR2 ASO or control (CON) ASO (5 or 15 mg/kg body weight) was subcutaneously injected in mice once a week (Figure 2(A)). At 24 weeks, we checked the mRNA expression of *Ddr1* and *Ddr2* in mice kidneys. DDR2 ASO suppressed the *Ddr2* mRNA expression (Figure 2(B)), but did not affect the *Ddr1* mRNA level (Figure 2(C)) in AS mice. Moreover, DDR2 ASO at 15 mg/kg, but not at 5 mg/kg, also significantly decreased the protein level of DDR2 in kidneys of AS mice (Figure 2(D-G)). These results indicated that DDR2 ASO especially at 15 mg/kg suppressed the DDR2 expression in AS mice. DDR2 ASO did not affect the DDR1 protein expression level, indicating the specificity of DDR2 ASO (Figure 2(D,E)).

### DDR2 ASO did not improve the kidney function in as mice

**Figure 3.**
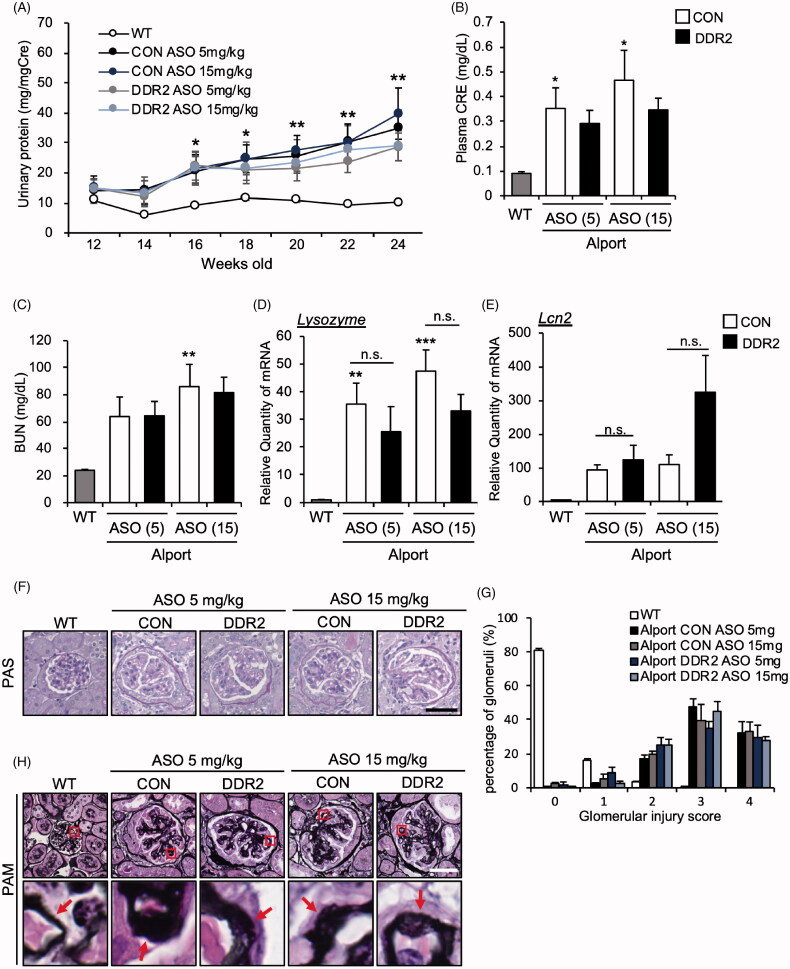
DDR2 ASO did not improve kidney function in Alport mice. (A) Urinary protein and creatinine were measured by Bradford and Jaffe’s method, respectively. Proteinuria score was calculated based on urinary protein and creatinine concentrations. *p<.05, **p<.01 vs WT, assessed by Dunnett’s test. (B) Serum creatinine level, and (C) blood urea nitrogen (BUN) were measured in 24-week-old WT, CON ASO- and DDR2 ASO-treated mice. Bars indicate the mean ± S.E. (n = 5–6). *p<.05, **p<.01 vs WT, assessed by Dunnett’s test. (D,E) Total RNA was isolated from kidney tissues of 24-week-old mice. Quantitative RT-PCR was performed to evaluate the expression of the indicated renal injury markers. The data were normalized to *Gapdh*. Bars indicate the mean ± S.E. (*n* = 5–6). **p<.01, ***p<.001 vs WT, assessed by Dunnett’s test. n.s., not significant. (F) Images of PAS-stained renal sections of 24-week-old mice are shown. (G) Glomerulosclerosis score was quantified from PAS-stained sections. (H) PAM staining of renal sections of 24-week-old mice was performed. *Lower panels*, enlarged view of the area boxed by the red square in upper panel. Red arrows indicate the GBM. Scale bars in F and H, 50 µM.

We investigated the effect of DDR2 ASO on kidney function by measuring renal parameters. Contrary to our expectation, DDR2 ASO did not improve the proteinuria in AS mice (Figure 3(A)). It also did not improve plasma creatinine and BUN (Figure 3(B,C)). We checked the mRNA expression of renal injury markers *Lysozyme* and lipocalin2 (*Lcn2*), which are highly induced in AS mice [4]. We found that the expression level of *Lysozyme* and *Lcn2* in AS mice were not significantly suppressed by DDR2 ASO (Figure 3(D,E)). We examined the renal pathology by PAS staining for glomerular injury, and assessed the injury score. The data revealed that high score of glomerular injury (3 to 4) was not alleviated by DDR2 ASO, indicating that the severity of glomerular injury in AS mice was not improved by DDR2 ASO treatment (Figure 3(F,G)). Furthermore, PAM staining showed that the thickening of glomerular basement membrane that is typically seen in AS mice glomeruli (ASO CON) was not inhibited by DDR2 ASO at 5 or 15 mg/kg (Figure 3(H)). Together, these results suggested that DDR2 ASO did not improve the kidney function in AS mice.

### DDR2 ASO did not affect the inflammatory and fibrosis gene expression in as mice

**Figure 4.**
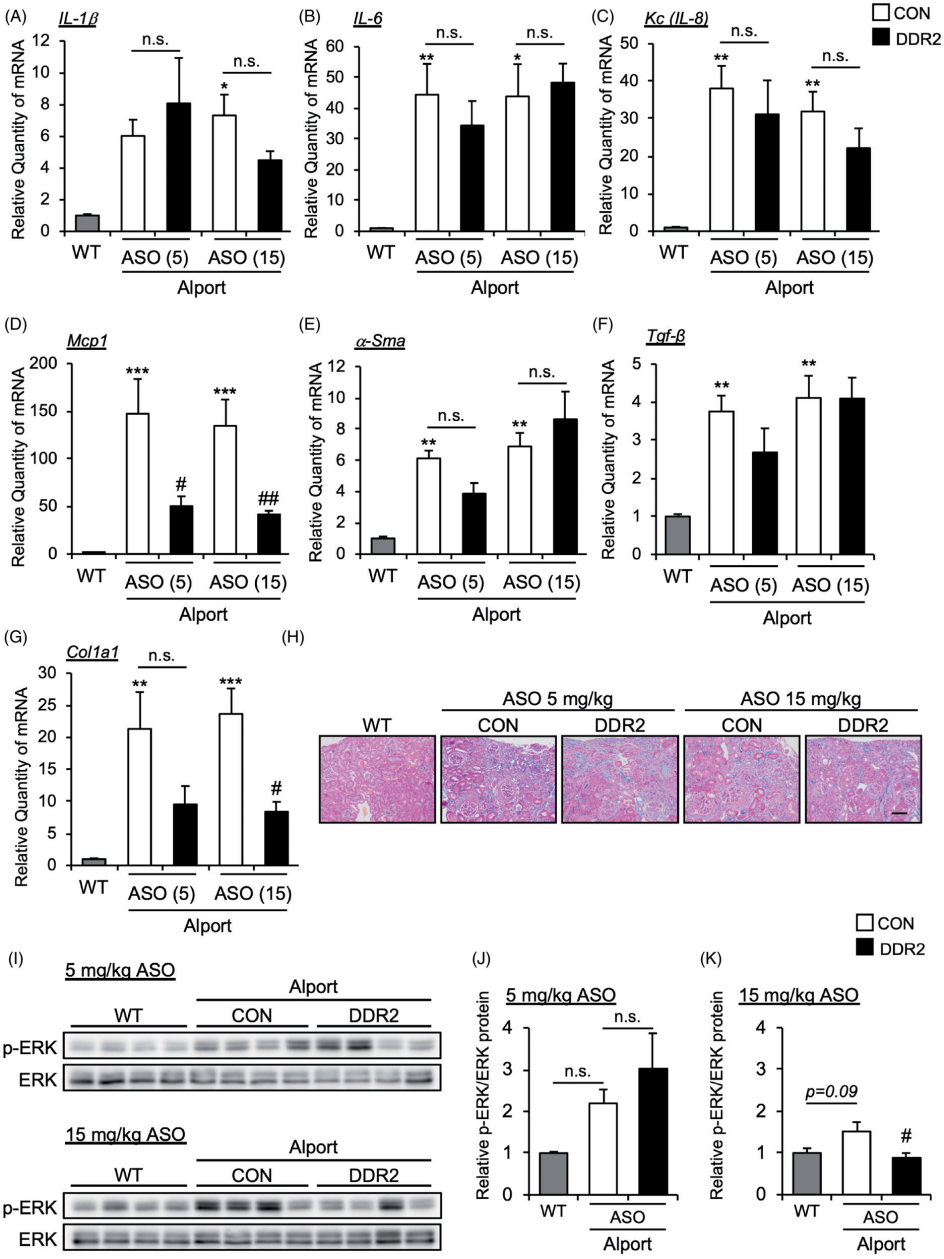
DDR2 ASO did not affect inflammatory and fibrosis gene expression in Alport mice. Total RNA was isolated from kidney tissues of 24-week-old mice. Quantitative RT-PCR was performed to evaluate the expression of the indicated (A-D) cytokines and (E-G) renal fibrosis markers. The data were normalized to *Gapdh*. Bars indicate the mean ± S.E. (*n* = 5–6). *p<.05, **p<.01, ***p<.001 vs WT; ^#^*p*<.05, ^##^*p*<.01 vs CON ASO, assessed by Dunnett’s test. (H) Masson-Trichrome staining of renal section of 24-week-old mice was performed. Scale bar, 100 µM. (I) Immunoblots of protein lysates from whole kidneys of WT and AS mice were probed with phosphorylated ERK (p-ERK) or ERK antibodies. (J,K) The p-ERK expression was quantified by multi gauge software and normalized with basal ERK. Bars indicate the mean ± S.E. (*n* = 4). ^#^*p*<.05 vs CON ASO, assessed by Dunnett’s test.

Next, we measured the mRNA expression of inflammatory cytokines in the kidney tissue of CON ASO- and DDR2 ASO-treated AS mice. The mRNA expression levels of *Il-1β*, *Il-6* and *Kc* (*Il-8* mouse homolog) were not changed in the kidneys of DDR2 ASO-treated AS mice compared with CON ASO-treated mice (Figure 4(A-C)). *Mcp1*, which is related to macrophage infiltration, is inhibited by DDR2 ASO (Figure 4(D)). We measured the expression of renal fibrosis markers *α-Sma*, *Tgf-β* and *Col1a1* in CON ASO- and DDR2 ASO-treated AS mice. Interestingly, DDR2 ASO suppressed the expression of *Col1a1* but not *α-Sma* and *Tgf-β* (Figure 4(E-G)). However, Masson-trichrome staining revealed that DDR2 ASO did not improve renal fibrosis (Figure 4(H)). These results suggest that although DDR2 ASO suppressed some cytokines and fibrosis marker, it did not improve the renal pathology of AS.

## Discussion

While the role of DDR1 in Alport syndrome has been studied, that of DDR2 is unknown. Here, we revealed that DDR2 mRNA and protein expression levels were significantly elevated in AS mouse model at 16 to 24 weeks old. To clarify its function, we knocked down DDR2 by administering antisense oligonucleotide in AS mice. DDR2 ASO suppressed the expression of *Mcp1*, an inflammatory cytokine and *Col1a1*, a fibrosis marker, but it did not improve AS pathology. DDR2 is expressed in mesenchymal cell, such as smooth muscle cells and fibroblasts [8,18]. It is known that fibroblasts differentiate into myofibroblasts by stimulating the fibrotic factor TGF-β and upregulating the expression of α-smooth muscle actin (α-SMA). Myofibroblasts increase the production of extracellular membrane such as type I collagen (COL1A1) and promote tissue fibrosis [19]. We checked the *Tgf-β*, *α-Sma* and *Col1a1* gene expression in kidney tissue in DDR2 ASO-injected AS model mice. DDR2 ASO reduced the *Col1a1* gene expression but did not change the *α-Sma* and *Tgf-β* gene expression (Figure 4(E-G)) in AS mice kidney. These results indicated that DDR2 ASO does not affect the differentiation from fibroblast to myofibroblast, but it inhibits the production of type I collagen (COL1A1) from myofibroblasts. Interestingly, a previous report showed that the deletion of *Ddr2* in mice attenuated the renal fibrosis induced by unilateral ureteral obstruction (UUO) [20]. It is possible that knockout rather than knockdown of *Ddr2* is effective in inhibiting fibrosis. Alternatively, the differences between UUO and AS mouse models may generate varied response to the blockade of *Ddr2*. The authors did not show the effect of *Ddr2* knockout on renal function after UUO.

We found here that DDR2 ASO did not improve AS pathology such as proteinuria and renal fibrosis. A possible mechanism for this lack of effect is the contribution of DDR1 in AS pathology. A previous report indicated that DDR1 expression is elevated in glomerulonephritis such as Goodpasture's syndrome, and *Ddr1* knockout improved renal pathology and suppressed the gene expression of inflammatory cytokine, renal injury and fibrosis markers such as type I collagen in mouse model of glomerulonephritis [10]. Moreover, *Ddr1* knockout improved the renal function in AS model mice (COL4A3 KO) [11,12]. These reports suggest that DDR1 is associated in the progression of AS pathology. Consistent with these previous reports, we also observed that *Ddr1* expression was slightly elevated as AS progresses. Because DDR2 ASO specifically downregulated *Ddr2* and did not affect *Ddr1* expression (Figure 2(B-C,D-E)), it may be possible that DDR1 contributed to the AS disease progression as indicated in previous studies mentioned above. So, we considered that not only *Ddr2* but also inhibition of *Ddr1* may be important for improvement of AS pathology. We observed that the mRNA level of *Ddr1* was only slightly elevated in AS mice kidneys (Figure 1(B)). The reason for this is unclear, but it may be that because of the absence of type IV collagen, a ligand of DDR1, in these AS mice, the *Ddr1* gene was not robustly activated. The mechanisms driving DDR1 expression and activation are not yet well defined [21].

The mechanism by which DDR2 ASO suppressed the gene expression of *Mcp1* and *Col1a1* is still unresolved. It is known that Mcp1 and Col1a1 mRNA expression levels are increased by MAPK signaling [22–24]. Moreover, activation of MAPKs ERK1/2 and p38 is associated with kidney disease, and inhibition of MAPK signaling ameliorates kidney injury [25–27]. It has also been reported that DDR2 activates MAPK signaling in various tissues [28–30]. Especially, DDR2 activates ERK2/SNAIL1 signaling stimulated by type I collagen and this axis is involved in the production of new type I collagen [31]. We checked the protein level of ERK1/2 in kidney tissue of AS model mice injected with DDR2 ASO, and found that ERK1/2 protein level was slightly reduced in 15 mg/kg DDR2 ASO-treated group compared with CON ASO (Figure (4(I-K)). This result suggests that slight inhibition of ERK1/2 by DDR2 ASO may suppress Mcp1 and Col1a1 mRNA expression. On the other hand, it was previously reported that DDR1, similar to DDR2, activates ERK signaling [32–35], indicating a similarity of DDR1 and DDR2 functions. Thus, further examination of the differences between DDR1 and DDR2 downstream signaling may lead to the discovery of new therapeutic targets of AS.
